# CD206-Targeted Liposomal Myelin Basic Protein Peptides in Patients with Multiple Sclerosis Resistant to First-Line Disease-Modifying Therapies: A First-in-Human, Proof-of-Concept Dose-Escalation Study

**DOI:** 10.1007/s13311-016-0448-0

**Published:** 2016-06-20

**Authors:** Alexey Belogurov, Konstantin Zakharov, Yakov Lomakin, Kirill Surkov, Sergey Avtushenko, Peter Kruglyakov, Ivan Smirnov, Gleb Makshakov, Curtis Lockshin, Gregory Gregoriadis, Dmitry Genkin, Alexander Gabibov, Evgeniy Evdoshenko

**Affiliations:** 1 M.M. Shemyakin and Yu.A. Ovchinnikov Institute of Bioorganic Chemistry, Russian Academy of Sciences, Moscow, Russia; 2 Institute of Fundamental Medicine and Biology, Kazan Federal University, Kazan, Tatarstan Russia; 3 Institute of Gene Biology RAS, Moscow, Russia; 4 OJSC Pharmsynthez, St. Petersburg, Russia; 5 Center of multiple sclerosis and AID of St. Petersburg City Clinical Hospital #31, St. Petersburg, Russia; 6 Pavlov First Saint-Petersburg State Medical University, St. Petersburg, Russia; 7 Xenetic Biosciences Inc., Lexington, MA USA; 8 Chemistry Department of Moscow State University, Moscow, Russia; 9 SBI “Center of Clinical Trials Management and Performance of Moscow Department of Healthcare”, Moscow, Russia

**Keywords:** Clinical trial, MRI, Myelin basic protein, Liposomes, Xemys, Mannose

## Abstract

Previously, we showed that CD206-targeted liposomal delivery of co-encapsulated immunodominant myelin basic protein (MBP) sequences MBP_46–62_, MBP_124–139_ and MBP_147–170_ (Xemys) suppressed experimental autoimmune encephalomyelitis in dark Agouti rats. The objective of this study was to assess the safety of Xemys in the treatment of patients with relapsing-remitting multiple sclerosis (MS) and secondary progressive MS, who failed to achieve a sustained response to first-line disease-modifying therapies. In this phase I, open-label, dose-escalating, proof-of-concept study, 20 patients with relapsing-remitting or secondary progressive MS received weekly subcutaneously injections with ascending doses of Xemys up to a total dose of 2.675 mg. Clinical examinations, including Expanded Disability Status Scale score, magnetic resonance imaging results, and serum cytokine concentrations, were assessed before the first injection and for up to 17 weeks after the final injection. Xemys was safe and well tolerated when administered for 6 weeks to a maximum single dose of 900 μg. Expanded Disability Status Scale scores and numbers of T2-weighted and new gadolinium-enhancing lesions on magnetic resonance imaging were statistically unchanged at study exit compared with baseline; nonetheless, the increase of number of active gadolinium-enhancing lesions on weeks 7 and 10 in comparison with baseline was statistically significant. During treatment, the serum concentrations of the cytokines monocyte chemoattractant protein-1, macrophage inflammatory protein-1β, and interleukin-7 decreased, whereas the level of tumor necrosis factor-α increased. These results provide evidence for the further development of Xemys as an antigen-specific, disease-modifying therapy for patients with MS.

**Electronic supplementary material:**

The online version of this article (doi:10.1007/s13311-016-0448-0) contains supplementary material, which is available to authorized users.

## Introduction

Multiple sclerosis (MS) is a chronic neurodegenerative disease with an evident autoimmune background resulting in inflammatory demyelination and axonal and neuronal injury [1]. MS, which was first described in 1868 [2], is one of the most common diseases of the nervous system. It affects people aged 20–40 years worldwide, although it has higher occurrence in women than in men and in those residing in northern than in southern latitudes. Despite its long history and the finding that immune cells rather than exogenous pathogens are responsible for MS development [3], the etiology of MS remains unclear.

Several treatment strategies for MS have been found to be moderately successful [4]. For example, the β-interferons and glatiramer acetate (GA) are disease-modifying therapies with an established history of efficacy and safety in clinical practice [5–7]. In addition, monoclonal antibodies binding to specific ligands have been found effective; these include natalizumab, which binds to α4 integrins [8]; daclizumab, which binds to CD25 [9]; and alemtuzumab, which binds to CD52 [10]. Natalizumab and alemtuzumab have been approved by the US Food and Drug Administration for the treatment of refractory MS, while daclizumab approval is very likely in the near future. Despite their effectiveness, however, these agents have been associated with serious adverse events (SAEs), significantly restricting their further application [4]. Novel, convenient oral therapies, including fingolimod [11], teriflunomide [12], and dimethyl fumarate [13], have shown efficacy and tolerability and have been approved for the treatment of patients with MS.

However, some patients remain refractory to these agents. This may be due to as yet unknown triggers of MS, together with high heterogeneity of this disease. Therefore, searching for novel, antigen-specific immunotherapeutic treatment options for MS is highly feasible [14]. For example, myelin basic protein (MBP), the structural component of the myelin membrane, is thought to be a primary target of the immune system during MS development [15]. Attempts have been made to induce tolerance toward MBP and its structural constituents [16–20], including MBP pulsing of dendritic cells [21]. In our previous studies using a newly designed MBP epitope library, we determined that MBP peptides 46–62, 124–139 and 147–170, but not 83-99, were the most immunodominant in terms of autoantibody responses in patients with MS when compared with healthy individuals and patients with other neurological diseases lacking an autoimmune background [22, 23]. Nasal administration of these MBP peptides suppressed protracted relapsing experimental allergic encephalomyelitis (EAE) in dark Agouti rats [24]. Further, selected immunodominant MBP peptides encapsulated into mannosylated liposomes were reported effective in the treatment of EAE [25]. Mannosylation of these liposomes was found critical for their therapeutic efficiency, as animals that received nonmannosylated peptide-loaded liposomes were unable to recover from the first EAE attack. The most reasonable explanation that was confirmed experimentally suggests that mannosylation of liposomes significantly enhances their uptake by dendritic cells via the CD206 receptor [25], resulting in immune system tolerance towards myelin antigens. The synergistic liposome-mediated effects of coencapsulated MBP peptides reduced overall disease course, resulting in moderate severity of attacks and faster recovery from exacerbations [25]. Preclinical studies showed that administration of the designed formulation, at doses largely exceeding those proposed for humans, did not induce significant AEs in animals. The aim of the present study was to explore the AE profile and tolerability of encapsulated MBP peptides in a cohort of patients with MS. Secondary outcomes were to evaluate the effects of these peptides on the clinical course of MS.

## Methods

### Study Design

**Fig. 1:**
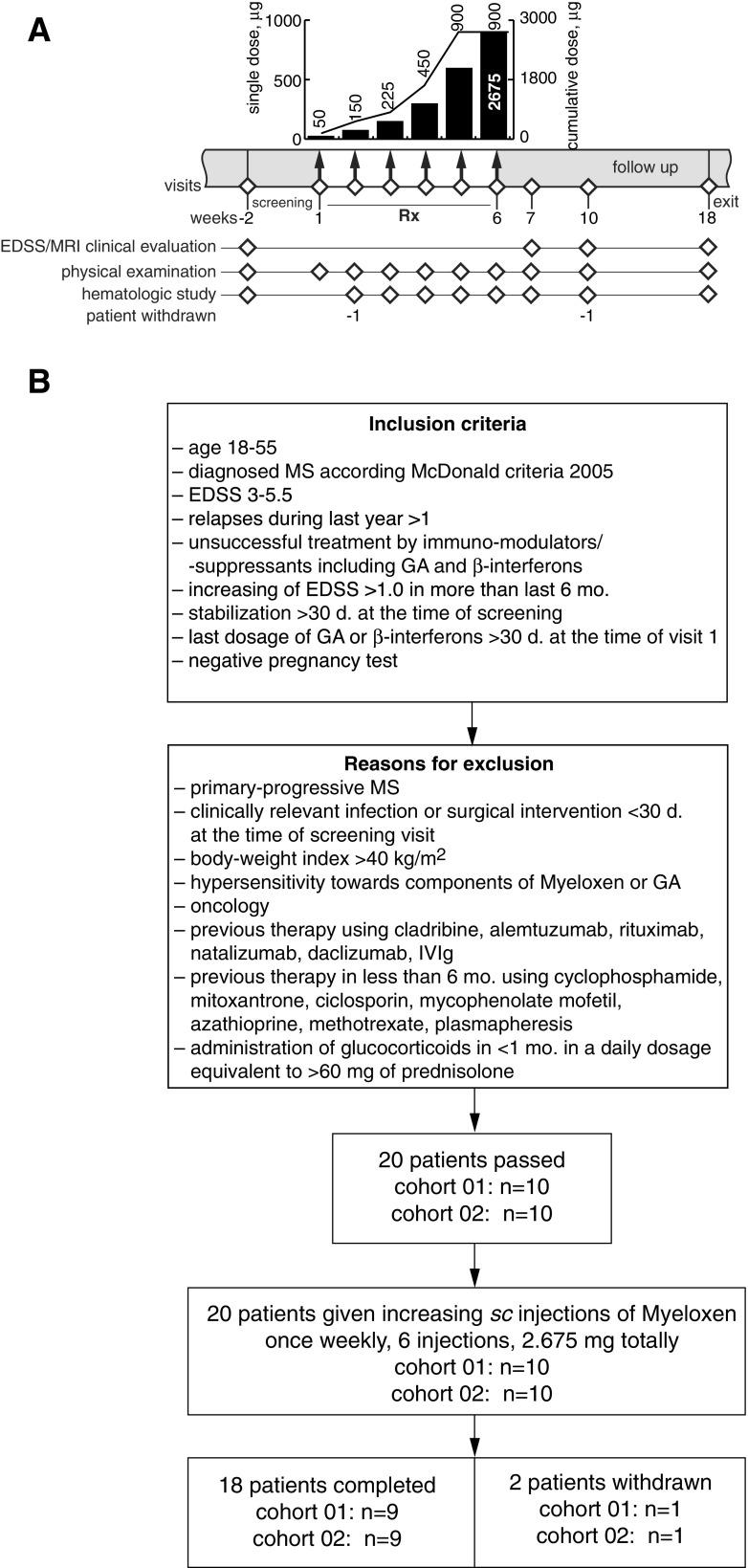
(A) Protocol schedule. Timeline showing major clinical and investigational elements of the study. (B) Subject allocation. Summary of screening outcome, treatment allocation, and study completion. Rx = treatment; EDSS = Expanded Disability Status Scale; MRI = magnetic resonance imaging; MS = multiple sclerosis; GA = glatiramer acetate; IVIg = intravenous immunoglobulin

This was a phase I, multicenter, open-label, dose-escalating safety, and proof-of-concept study of the oligopeptides MBP_46–62_, MBP _124–139,_ and MBP _147–170_ coencapsulated in CD206-targeted small monolammelar liposomes (Xemys; Pharmsynthez, St. Petersburg, Russia [25]) in patients with relapsing-remitting (RRMS) or secondary progressive MS with superimposed relapses (SPMS) who failed to achieve sustained responses to first-line disease-modifying therapies (FASEMS). The FASEMS clinical trial schedule is summarized in Fig. 1A. Patients received 6 weekly subcutaneous injections, on the same day each week, of Xemys at doses ascending from 50 μg to 900 μg. After the last injection, patients were followed up for 12 weeks.

The primary endpoint was the safety of Xemys, as determined by the frequency and severity of adverse events (AEs) and SAEs. To ensure patient safety, the patients were divided into 2 cohorts. Dose-limiting toxicities (DLT) and dose adjustments were assessed in the first cohort; doses in the second cohort were limited to the highest first-cohort dose not associated with DLT. If there were no DLTs in the first cohort, the dosing regimen would remain unaltered in the second cohort.

Secondary clinical endpoints included the number of relapses during the study period and Expanded Disability Status Scale (EDSS) score at the end of the trial. Secondary magnetic resonance imaging (MRI) endpoints included the number of gadolinium-enhancing T1 lesions and the total number of lesions in T2 and fluid-attenuated inversion recovery (FLAIR) sequences. Laboratory endpoints included the concentrations of pro- and anti-inflammatory cytokines. The study was approved by the Russian Ministry of Health, the Central Ethics Commission, and all local ethic committees, and was conducted in full compliance with the Declaration of Helsinki, International Conference on Harmonisation’s Good Clinical Practice, and appropriate local legislation.

### Subjects

Subjects were screened 2 weeks before enrollment into the treatment phase of the study (Fig. 1B). The trial involved patients with RRMS or SPMS with superimposed relapses, defined as previously described [26]. Subjects were included if they were aged 18–55 years, had an EDSS from 3 to 5.5 and a ≥ 1.0 increase during the previous 6 months, and had > 1 relapse during the previous year [27]. Subjects also had to be stable for > 30 days at the time of screening and to have not received treatment with GA or β-interferons for > 30 days at the time of visit 1. Female subjects had to have negative pregnancy tests.

Subjects were excluded if they had primary progressive MS; clinically relevant infection or surgical intervention < 30 days before the screening visit; contraindications to MRI scanning, including hypersensitivity toward gadolinium; a body mass index > 40 kg/m^2^; or hypersensitivity toward components of a test item (egg phosphatidylcholine, monomannosyl dioleyl glycerol, α-tocopherol, or lactose) or GA. Subjects were also excluded if they had liver decompensation; heart diseases; tuberculosis in anamnesis; significantly abnormal hematological or biochemical parameters; oncological diseases; previous therapy with cladribine, alemtuzumab, rituximab, natalizumab, daclizumab, or intravenous immunoglobulin; treatment with any disease-modifying therapy during the previous 6 months, including cyclophosphamide, mitoxantrone, ciclosporin, mycophenolate mofetil, azathioprine, methotrexate, or plasmapheresis; or had been administered glucocorticoids within the last month at a daily dosage equivalent to > 60 mg of prednisolone.

The study was authorized by the Russian Public Health Ministry #930 (FASEMS-01/01) issued on 28 April 2012. All patients provided written informed consent at enrollment after discussion of the study with investigators including possible alternative treatment options. Details of full medical history and MS pathogenesis were collected.

### Procedures

Lyophilized Xemys consists of equimolar amounts of lyophilized, chemically synthesized MBP peptides 46–62 (GGDRGAPKRGSGKDSHH), 124–139 (GFGYGGRASDYKSAHK), and 147–170 (QGTLSKIFKLGGRDSRSGSPMARR) (total 0.45 mg) encapsulated in small unilamellar liposomes prepared from egg phosphatidylcholine and monomannosyl dioleyl glycerol with the addition of α-tocopherol and lactose (total 125 mg). Each dose was rehydrated in 1.0 ml sterile water immediately before administration. The starting dose, consisting of 0.05-mg peptides, was chosen as the minimum to detect any unpredictable AEs without significant risk to the patients. Patients received weekly subcutaneous injections of Xemys at escalating doses over 6 weeks of 50 μg, 150 μg, 225 μg, 450 μg, 900 μg, and 900 μg, yielding a total dose of 2.675 mg.

Patients were followed-up 1, 4, and 12 weeks after administration of the last dose, corresponding to study weeks 7, 10, and 18, respectively. AEs were monitored by 12-lead electrocardiography, hematology, and laboratory tests, and scored according to the Common Terminology Criteria for Adverse Events version 4.0. Any SAEs deemed “certainly” or “likely” due to the study drug were considered DLTs, with the next lowest dose level considered the maximum tolerated dose. Patients also underwent complete neurological examinations during each study visit. An MS *exacerbation* was defined as a new worsening of neurological function lasting for > 24 h that was unrelated to other comorbidities.

EDSS was determined at baseline (week 2) and at all follow-up visits. Patients underwent MRI scans, including T1-weighted axial scans with and without gadolinium, proton density axial, T2-weighted axial, T2-weighted sagittal, and FLAIR sequence axial images, at baseline and at follow-up visits at weeks 7, 10, and 18. Scans were performed with Philips, Amsterdam, Netherlands Integra 1.5T, Magnetom Avanto 1.5T, and GE Medical Systems, Milwaukee, WI, USA Signa 1.5T scanners.

Serum samples for cytokine analysis were collected at baseline and during all follow-up visits. The profiles of 17 cytokines and chemokines were determined using a multiplexed fluorescent magnetic bead-based immunoassay (Bio-Rad Laboratories, Berkeley, CA, USA), according to the manufacturer’s instructions. These 17 cytokines and chemokines included interleukin (IL)-1β, IL-2, IL-4, IL-5, IL-6, IL-7, IL-8, IL-10, IL-12 (p70), IL-13, IL-17A, granulocyte colony-stimulating factor, granulocyte macrophage colony-stimulating factor, interferon-γ, monocyte chemoattractant protein-1 (MCP-1/CCL2), macrophage inflammatory protein (MIP-1b/CCL4), and tumor necrosis factor (TNF)-α.

### Statistical Analysis

Demographic data, baseline characteristics, safety and tolerability variables, and other parameters under investigation were calculated using descriptive statistics. Safety and tolerability were assessed in patients who received at least 1 dose of the studied substance. AEs were grouped by dose and classified by MedDRA system organ classes and preferred terms, with severity classified by Common Terminology Criteria for Adverse Events version 4.0. Secondary endpoints were analyzed in patients who received at least 1 dose of the studied substance and underwent at least 1 assessment. The normality of the data was determined using Kolmogorov–Smirnov tests; all datasets were non-normally distributed. Changes from baseline in the number of MRI lesions were assessed by analysis of variance. Mann–Whitney *t* tests were used to compare between-group variables and the Wilcoxon signed rank test for within-group variables. All tests were two sided, and *p*-values < 0.05 were considered significant. All statistical analyses were performed with SPSS (IBM, Armonk, NY, USA) and GraphPad Prism 6.0 (GraphPad Inc., La Jolla, CA, USA).

## Results

Between April 2013 and July 2014, 20 patients with RRMS or SPMS matching all criteria were directly recruited into the trial at 4 clinical centers in the Russian Federation. Baseline characteristics of patients with MS are listed in Table 1. Inclusion and exclusion criteria, as well as the numbers of patients screened and enrolled in the study, are summarized in Fig. 1B. Of the 20 patients, 16 (80%) had RRMS and 4 (20%) had SPMS with relapses. At baseline, 3 (15%) patients had mild disability according to EDSS score (3.0), and 17 (85%) had moderate disability (3.5–5.5) (Table 2). Nineteen patients received all 6 weekly doses of encapsulated MBP peptides (total 2.675 mg) (Fig. 1A). One patient who received all 6 doses of Xemys discontinued from the study after treatment period at week 6 (his own decision); 1 patient received only the first 50-μg dose and chose to discontinue after 1 week (his own decision).

**Table 1:** Baseline characteristics of patients with multiple sclerosis

Characteristic	Variable
*n*	20
Age (years)^*^	37.6 ± 9.9 (24–53)
Sex^†^	9–11 (45%)
Weight (kg)^*^	69.7 ± 15.8 (44–105.5)

^*^Average ± SD (range)

^†^Female – male (female %)

**Table 2:** Multiple sclerosis (MS) anamnesis

Anamnestic criterion	Variable (*n* = 20)
MS type	
Relapsing-remitting	16 (80%)
Secondary progressive	4 (20%)
Baseline EDSS scores	
3.0	3 (15%)
3.5	3 (15%)
4.0	8 (40%)
4.5	2 (10%)
5.0	2 (10%)
5.5	2 (10%)
First signs of MS^*^	10.4 ± 6.8 (0.9–25.8)
MS diagnosis^*^	5.0 ± 3.6 (0.6–13.3)
MS exacerbations during the last:^†^	
1 year	2.0 (1–3)
2 years	3.0 (1–6)

Data are presented as *n* (%) unless otherwise indicated

^*^Average ± SD, min–max (years)

^†^Median (min–max)

EDSS = Expanded Disability Status Scale

As no patient experienced a DLT during treatment, an maximum-tolerated dose was not reached, making it likely to be > 900 μg per week. Eight patients (40%) experienced 16 AEs (Table 3), with 11 events in 5 (25%) patients regarded as related to the Xemys injections. No SAEs, serious drug reactions, or deaths occurred during the study. Of the 16 AEs, 13, in 6 (30%) patients, were regarded as grade 1, and 3 AEs, in 2 (10%) patients, were regarded as grade 2 (Table 4). No AE met the seriousness criteria of International Conference on Harmonisation E6. All drug-related AEs were grade 1 in severity, except for diarrhea, which was grade 2 (Table 5). All AEs resolved without treatment and did not require interruption or discontinuation of the investigational drug.

**Table 3:** Overview of adverse events (AEs)

Total (*n* = 20)*	8 (40%)/16
Serious AEs	0
AE, drug related	5 (25%)/ 11
AE, led to drug interruption	0
AE, required therapy	2 (10%)/ 2
Suspected unexpected serious adverse reactions	0
Deaths	0

^*^Data are presented as *n* (%)/c, where *n* = number of subjects, % = part of subjects with c = number of AEs

**Table 4:** Adverse events by MedDRA preferred term and by Common Terminology Criteria for Adverse Events (CTCAE) severity grades

System organ class/preferred term	Grade by CTCAE 4.0*	
	1 grade (mild)	2 grade (moderate)
Total	6 (30%)/13	2 (10%)/3
Gastrointestinal	0	2 (10%)/2
Pain in upper abdomen	0	1 (5%)/1
Diarrhea	0	1 (5%)/1
General disorder and administration site conditions	4 (20%)/10	0
Weakness	1 (5%)/2	0
Injection site reaction	4 (20%)/8	0
Infection and infestation	2 (10%)/2	0
Rhinitis	2 (10%)/2	0
Nervous system disorders	1 (5%)/1	0
Spasm of muscles of lower extremities	1 (5%)/1	0
Vascular disorders	0	1 (5%)/1
Essential hypertension	0	1 (5%)/1

^*^Data are presented as *n* (%)/c, where *n* = number of subjects, % = part of subjects with c = number of adverse events

**Table 5:** Adverse events by MedDRA preferred term and relationship to study drug

System organ class/preferred term	Relationship (*n* = 20)*		
	Not related	Possible	Likely
Total	3 (15%)/5	1(5%)/1	5 (25%)/10
Gastrointestinal	1 (5%)/1	0	1 (5%)/1
Pain in upper abdomen	1 (5%)/1	0	0
Diarrhea	0	0	1 (5%)/1
General disorder and administration site conditions	0	1(5%)/1	4 (20%)/9
Weakness	0	1(5%)/1	1 (5%)/1
Injection site reaction	0	0	4 (20%)/8
Infection and infestation	2 (10%)/2	0	0
Rhinitis	2 (10%)/2	0	0
Nervous system disorders	1 (5%)/1	0	0
Spasm of muscles of lower extremities	1 (5%)/1	0	0
Vascular disorders	1 (5%)/1	0	0
Essential hypertension	1 (5%)/1	0	0

^*^Data presented as *n* (%)/c, where *n* = number of subjects, % = part of subjects with c = number of adverse events

The most common AE was local reaction at the site of injection, which was observed 8 times in 4 (20%) patients. Most injection site reactions occurred at administration of submaximal (0.45 mg) and maximal (0.9 mg) doses of Xemys; all resolved within 24 h without treatment. Rhinitis occurred twice in 2 patients (10%) each and general weakness twice in 1 (5%) patient. Other AEs occurred only once in single patients. Xemys did not have any effect on laboratory safety tests, vital signs (body temperature, heart rate, respiration rate and blood pressure), results of physical examination, or electrocardiography parameters (heart rate, PR interval, QRS duration, and QT interval). In general, the administered doses of Xemys were considered safe and tolerable.

**Fig. 2:**
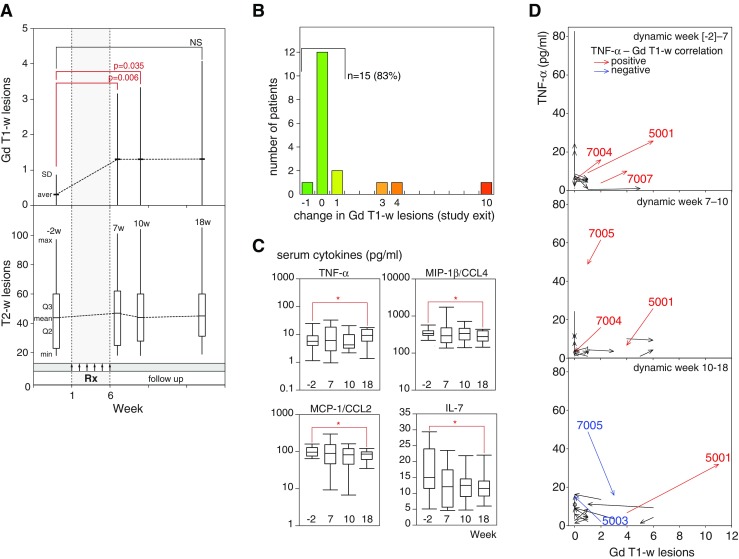
(A) Cumulative change in mean number of new gadolinium-enhancing and T2-weighted (w) lesions. (B) Change in number of gadolinium (Gd)-enhancing lesions at study exit (18 weeks). (C) Concentrations of serum cytokines at screening (–2 weeks), 7, 10, and 18 weeks. The bold lines represent the medians and the boxes represent interquartile ranges. Bars represent 95% confidence intervals. Statistically significant differences (*p* < 0.05) are indicated. (D) Vector plots showing changes in serum tumor necrosis factor (TNF)-α concentrations and numbers of gadolinium-enhancing lesions in studied patients. Positive and negative correlations between TNF-α level and number of gadolinium-enhancing lesions are shown in red and blue, respectively. Numbers represent patient identifications. Aver = average; Q = quartile; min = minimal value; max = maximal value; MIP = macrophage inflammatory protein; MCP = monocyte chemoattractant protein; IL = interleukin

Patients underwent MRI scans at baseline (week –2) and after treatment, at weeks 7, 10, and 18, using T1-weighted (with and without contrast), T2, and FLAIR regimens. Nineteen patients were evaluated at baseline and week 10, and 18 patients at weeks 7 and 18 (Table 6, Fig. 2A). At baseline, 16 (84%) patients had no active gadolinium-enhancing lesions. By week 7, however, active lesions were detected in 10 (56%) patients, and at last follow-up, 8 (33.7%) patients had active lesions. Although a trend towards an increasing number of gadolinium-enhancing lesions was detected, per subject-specific analysis of MRI results at time of study exit showed that 83% of patients had 0±1 new lesions, with only 3 patients (17%) having > 1 new lesion (Fig. 2B). To assess changes in the number of MRI lesions in comparison with baseline, ANOVA was performed, with the dependent variable being the rank of the number of MRI lesions (Table 7). All datasets were non-normally distributed. Relative to baseline, the increases in the number of active gadolinium-enhancing lesions were statistically significant at weeks 7 and 10 (*F* = 3.015, *p* = 0.038), but not at week 18 (*p* > 0.05). At last follow-up, 16/19 (85%) patients were relapse-free, and EDSS worsened in 21% and improved in 10% of patients. The disease histories, and clinical and MRI outcomes of individual patients are summarized in Table 8.

**Table 6:** Magnetic resonance imaging (MRI) lesions by visits

Visit	MRI regime	*n*	MRI Lesions				
			Mean^*^	Median	Min-max	Q 25%	Q 75%
Baseline	T1	19	0.3 ± 0.6	0.0	0–2	0.0	0.0
	T2		45.2 ± 20.0	44.0	18–97	23.0	60.0
	FLAIR		46.9 ± 20.4	44.0	21–96	24.0	62.0
Week 7	T1	18	1.3 ± 1.9	1.0	0–6	0.0	2.0
	T2		47.7 ± 21.7	47.0	18–101	25.0	62.0
	FLAIR		49.2 ± 22.1	47.0	23–100	25.0	67.0
Week 10	T1	19	1.3 ± 2.0	0.0	0–6	0.0	2.0
	T2		47.5 ± 22.0	44.0	18–104	28.0	60.0
	FLAIR		48.5 ± 21.6	44.0	23–103	28.0	62.0
Week 18	T1	18	1.3 ± 2.8	0.0	0–11	0.0	1.0
	T2		48.2 ± 22.2	45.0	19–105	31.0	60.0
	FLAIR		49.6 ± 22.0	46.0	24–104	31.0	62.0

^*^Average ± SD

FLAIR = fluid-attenuated inversion recovery

**Table 7:** Gadolinium-enhancing T1 magnetic resonance imaging lesions by visits (analysis of variance)

Weeks	Marginal mean for visits*	95% CI	Pared comparison			F^‡^	*p* ^‡^
			Difference of means^*,†^	*p*	95% CI		
Baseline	2.0 ± 0.2	(1.5-2.4)				3.015	0.038
Week 7	2.8 ± 0.2	(2.4-3.3)	–0.9 ± 0.3	0.006	(–1.50 to –0.27)		
Week 10	2.6 ± 0.2	(2.2-3.0)	–0.7 ± 0.3	0.035	(–1.25 to –0.05)		
Week 18	2.4± 0.2	(2.0-2.9)	–0.5 ± 0.3	0.115	(–1.11 to 0.12)		

^*^Data are presented ± SE

^†^Difference of means between baseline and weeks 7, 10, and 18

^‡^For “visit” factor assessment

CI = confidence interval

**Table 8:** Patients' history, magnetic resonance imaging, and clinical outcome measures at study exit

ID	Diagnosis	Duration of disease (years)	Relapses during last 12 months	Previous treatment*	Cumulative number of new Gd+ lesions	Cumulative number of new T2 lesions	Actual EDSS delta	Relapse during study
7001	RRMS	7.8	3	IFN-β	0	0	0	N
7002	RRMS	9.9	2	IFN-β	0	0	–0.5	N
7004	RRMS	3.9	2	IFN-β	1	2	–1	N
7005	RRMS	1.5	2	IFN-β	3	2	0	N
7006	RRMS	1.5	1	IFN-β	0	2	0.5	Y
7007	RRMS	6.5	2	GA	-1	10	1	Y
7008	SPMS	0.6	1	IFN-β	0	0	0	N^a^
5001	RRMS	6.0	1	IFN-β	10	0	0	N
5002	SPMS	9.9	2	IFN-β	0	0	0	N
5003	SPMS	4.3	1	IFN-β	0	0	0	N
4001	RRMS	1.4	1	GA	0	13	0	N
4002	SPMS	9.4	2	IFN-β	0	9	0	N
4003	RRMS	2.9	1	IFN-β	0	0	0	N
4004	RRMS	4.7	1	IFN-β	0	2	0	N
4005	RRMS	7.2	3	IFN-β	0	1	0	N
4006	RRMS	0.7	2	IFN-β	0	1	0.5	N
4007	RRMS	2.6	2	IFN-β	0	0	0	N
2001	RRMS	13.3	2	GA	1	1	0.5	N
2002	RRMS	3.2	3	GA/IFN-β	4	8	0	Y

^*^GA – glatiramer acetate

^a^The last visit is Day 64, Day 120 is missing

EDSS = Expanded Disability Status Scale; RRMS = relapsing-remitting multiple sclerosis; IFN = interferon; GA = glatiramer acetate; SPMS = secondary progressive multiple sclerosis

To analyze the immunological consequences of Xemys administration, the concentrations of 17 serum cytokines and chemokines were analyzed at follow-up time points (Fig. 2C). Compared with baseline, MCP-1, MIP-1β, and IL-7 concentrations were significantly lower and TNF-α was significantly higher at study exit (week 18).

## Discussion

This study showed that subcutaneous administration of Xemys was safe and well tolerated in patients with MS who had previously failed to achieve sustained disease control following treatment with GA or β-interferons. The AEs related to Xemys were of mild or moderate severity and occurred mainly after treatment with submaximal and maximal doses of Xemys. These AEs were self-limiting, required no concomitant medication, and did not cause abnormalities in blood tests or other safety measures. Taken together, these findings showed that once-weekly Xemys has a relatively good safety profile.

Patients included in the FASEMS trial have experienced significant disease activity, despite previous first-line disease-modifying treatment. Importantly, 12 weeks after the end of Xemys administration, 7 patients (37%) showed no evidence of disease activity, and 16 (85%) were relapse-free. Although EDSS levels and the numbers of T2-weighted lesions and new gadolinium-enhancing lesions on MRI in comparison with baseline were statistically unchanged at study exit, a statistically significant increase in the number of active gadolinium-enhancing lesions on weeks 7 and 10 in comparison with baseline was detected. Here we emphasize that all patients included in this study had experienced > 1 relapse during the previous year, had progressing EDSS, and at the same time had disease stabilization for at least 30 days before screening. Therefore, the number of contrast-enhancing lesions may have had a natural tendency to increase during the course of the study. The lack of a placebo group reasoned by approved study design did not allow comparative monitoring of disease progression, including the number of gadolinium-enhancing lesions, in the absence of therapeutic intervention. Finally, the statistically significant increase in the number of gadolinium-enhancing lesions was assessed only in a small group of patients who experienced relapse. Thus, the appearance of these lesions was not likely associated with treatment, although this possibility cannot be completely excluded.

Our preliminary data also suggest that there were no consistent trends in serum cytokine profiles. However, the concentrations of MCP-1, MIP-1β, and IL-7 were significantly lower after treatment that at baseline. Immunologically, Xemys administration is thought to restrict monocyte cell trafficking. Although a classical proinflammatory cytokine TNF-α has been previously shown to have potent beneficial effects in autoimmune neurodegeneration [28], reported herein increase in concentration of serum TNF-α in treated patients with MS had likely no protective input. Despite the absence of a distinct link between changes of the number of gadolinium-enhancing lesions and dynamic of serum level of TNF-α, a rather positive correlation was observed (Fig. 2D).

In conclusion, this phase I trial showed that once-weekly subcutaneous Xemys for 6 weeks, at a cumulative dose of 2.675 mg, was safe and well tolerated by patients with RRMS and SPMS. Preclinical and clinical data warrant the further development of Xemys as an antigen-specific disease-modifying therapy for patients with MS.

## Electronic supplementary material

Below is the link to the electronic supplementary material.

ESM 1 (PDF 1225 kb)
